# Islet1 and Brn3 Expression Pattern Study in Human Retina and hiPSC-Derived Retinal Organoid

**DOI:** 10.1155/2019/8786396

**Published:** 2019-12-10

**Authors:** Ziming Luo, Chaochao Xu, Kaijing Li, Bikun Xian, Yuchun Liu, Kang Li, Ying Liu, Huifeng Rong, Mingjun Tang, Dongpeng Hu, Sijing Yang, Meifang Ye, Xiufeng Zhong, Jian Ge

**Affiliations:** State Key Laboratory of Ophthalmology, Guangdong Provincial Key Laboratory of Ophthalmology and Visual Science, Zhongshan Ophthalmic Center, Sun Yat-sen University, Guangzhou, Guangdong, China 510060

## Abstract

This study was conducted to determine the dynamic Islet1 and Brn3 (POU4F) expression pattern in the human fetal retina and human-induced pluripotent stem cell- (hiPSC-) derived retinal organoid. Human fetal eyes from 8 to 27 fetal weeks (Fwks), human adult retina, hiPSC-derived retinal organoid from 7 to 31 differentiation weeks (Dwks), and rhesus adult retina were collected for cyrosectioning. Immunofluorescence analysis showed that Islet1 was expressed in retinal ganglion cells in the fetal retina, human adult retina, and retinal organoids. Unexpectedly, after Fwk 20, Brn3 expression gradually decreased in the fetal retina. In the midstage of development, Islet1 was detected in bipolar and developing horizontal cells. As the photoreceptor developed, the Islet1-positive cone precursors gradually became Islet1-negative/S-opsin-positive cones. This study highlights the distinguishing characteristics of Islet1 dynamic expression in human fetal retina development and proposes more concerns which should be taken regarding Brn3 as a cell-identifying marker in mature primate retina.

## 1. Introduction

Islet1, also known as ISL1, is a LIM-homeodomain transcription factor that plays critical roles in differentiation, cell specification, and phenotype maintenance of horizontal cells as well as cholinergic amacrine and ganglion cells in the retina of different species including fish, reptiles, birds, amphibians, chickens, and mammals. Moreover, numerous studies have revealed that ISL1 plays a key role in multiple tissue types, such as the heart [1], kidneys [2], skeletal muscle, endocrine organs [3], and nervous system [4]. Additionally, previous studies suggested that ISL1 is highly expressed in gastric and breast cancer [5] and is associated with advanced tumor invasion, proliferation, migration, tumor stage, tumor size, metastasis, and poor overall survival. ISL1, as a crucial transcription factor, is required for retinal neuroblast differentiation during human retinogenesis. Various previous studies also demonstrated Islet1 dynamic expression shows a specific temporal and spatial pattern in the retina of multiple animal models [6–10]. However, the detailed Islet1 expression pattern during human retinal development remains unclear.

Since 2012 [11], studies using retinal organoids derived from human embryonic stem cells (hESCs) and human-induced pluripotent stem cells (hiPSCs) have provided insight into developmental biology research, disease modeling, and stem cell replacement therapy. As the production of retinal organoids for studying differentiation has increased [12], this approach has begun to replace animal models because of the lower cost, fewer ethical concerns, and humanization properties.

An essential question regarding retinal organoids is whether they mimic human fetal eye development. If so, Islet1 may be useful as an indicator for determining the developmental process of retinal organoids compared to the human fetal retina.

To evaluate Islet1 dynamic expression in human fetal retina and hiPSC-derived retinal organoid development, we characterized subsets of Islet1-positive cells based on morphological features and performed coimmunostaining with specific markers of retinal neuron subtypes. We determine the expression profile of Islet1 during human fetal retina development and examined variations in the developmental process in retinal organoids. During the study on Islet1, Brn3 showed distinguished express pattern from a rodent model. Therefore, the Brn3/Pou4f family of transcription factors, well-known markers of RGC, is also investigated in the present study.

## 2. Methods

### 2.1. Ethics Statement and Tissue Collection

Human fetal eyes ranged in age from 8 to 27 fetal weeks (Fwk) and were obtained from legal routine therapeutic abortions at the Third Affiliated Hospital, Sun Yat-sen University. Fetal age was determined by eye size and foot length. The human adult retina slices were collected by the pathology department of Zhongshan Ophthalmic Center, from the samples for pathology examination and choosing the peripheral normal retina. The retinoblastoma tissue was collected by Prof. Rong Lu. Rhesus slices were collected from the control eyes of a previous study [13]. All samples were collected with patient consents and in accordance with protocols approved by the institutional review boards at Zhongshan Ophthalmic Center, SYSU (approval number: 2013PRLL0029). Eyes for immunofluorescence (IF) and morphologic analysis were fixed in 4% (2–12 h) paraformaldehyde in 0.1 M phosphate-buffered saline (PBS). Only sections adjacent to the fovea were used for immunofluorescence.

### 2.2. Retinal Organoids

The retinal organoids were differentiated, as described previously [12], from the BC1-GFP hiPSC line (gifted by Prof. Linzhao Cheng, Johns Hopkins University School of Medicine, Baltimore, MD, USA) and the SB hiPSC line (CA4002106; Cellapy Bio, Beijing, China). The day on which embryonic bodies (EBs) were formed was considered as Day 0, and the retinal organoid was cultured in suspension after Day 28 (differentiation week 4, Dwk 4).

### 2.3. Fixation and Sectioning

Human fetal eyeballs < Fwk 15 were fixed with 4% paraformaldehyde in 0.1 M PBS (pH 7.2–7.3) for 2–4 h according to the size of the eyeballs at room temperature (24-26°C). In elder eyes, the anterior segment (including the cornea, iris, and lens) and vitreous were removed to obtain posterior cups for fixation. Residual eyecups were fixed overnight at 4°C. Retinal organoids were collected and fixed in 4% paraformaldehyde for 0.5 h.

Next, fetal eye tissues and retinal organoids were rinsed in 0.1 M PBS at room temperature to remove residual fixative and cryoprotected with a sucrose gradient (%, wt/vol) in phosphate buffer (6.25% for 1 h, 12.5% for 2 h, and 25% overnight at 4°C). Subsequently, the samples were embedded in optimal cutting temperature (OCT) compound (OCT: 25% sucrose = 1 : 2). A Leica CM1850 cryostat (Wetzlar, Germany) was used to produce serial sections at thickness of 10 *μ*m (retinal organoid) or 14 *μ*m (fetal retina), which were collected onto SuperFrost plus slides (Citotest, Nanjing, China).

### 2.4. Immunofluorescence and Hematoxylin-Eosin Staining

OCT compound was removed by a 15 min incubation in 37°C PBS, and cryosections were incubated in permeabilization and blocking solution (PB) containing 0.2% (vol/vol) Triton X-100 and 10% (wt/vol) normal donkey serum in PBS for 1 h at room temperature. Primary antibodies were incubated with the sections at 4°C overnight. The following primary antibodies were diluted in 2% (wt/vol) donkey serum, 0.04% (vol/vol) Triton X-100 in PBS: anti-Islet1 antibody (mouse, 1 : 200, Abcam, Cambridge, UK), anti-MCM2 antibody (rabbit, 1 : 200, Abcam), anti-VSX2 antibody (sheep, 1 : 500, Millipore, Billerica, MA, USA), anti-Brn3 antibody (goat, 1 : 200, Santa Cruz Biotechnology, Dallas, TX, USA), anti-Brn3a antibody (mouse, 1 : 20, Santa Cruz Biotechnology, Dallas, TX, USA), anti-RPBMS antibody (rabbit, 1 : 200, Abcam), anti-HuD antibody (mouse, 1 : 100, Santa Cruz Biotechnology), anti-AP2*α* antibody (goat, 1 : 200, Abcam), anti-Recoverin antibody (rabbit, 1 : 500, Abcam), anti-Rhodopsin antibody (mouse, 1 : 200, Abcam), anti-Ki67 (rabbit, 1 : 50, Boster, Wuhan, China), anti-S-opsin (rabbit, 1 : 5000), and anti-L/M-opsin (rabbit, 1 : 5000), which were donated by Jeremy Nathans (Department of Molecular Biology and Genetics, Neuroscience, and Ophthalmology, the Johns Hopkins University School of Medicine).

Primary antibody staining was followed by three washes with 1x PBS. Subsequently, cryosections were incubated for 1 h at room temperature with secondary antibodies that included the corresponding species-specific Alexa Fluor-488-, Alexa Fluor-555-, and Alexa Fluor-657-conjugated antibodies (1 : 500; Gibco, Grand Island, NY, USA). Next, 2 *μ*L ProLong® Gold Antifade Reagent with DAPI was applied 10 minutes at room temperature to counterstain the nuclei. Subsequently, all samples were rinsed in PBS and mounted in Vectashield and coverslipped. H&E staining was performed as described previously [14, 15].

### 2.5. Image Acquisition and Processing

Images were acquired on an Olympus BX53 microscope (Tokyo, Japan) and Zeiss (Oberkochen, Germany). Confocal images were acquired with a Zeiss LSM 510 confocal microscope. Immunofluorescence intensity was evaluated by ImageJ (NIH, Bethesda, Maryland), and for statistical comparisons, values were subjected to a two-tailed Student's *t* test Prism Software Version 7 (GraphPad Software, Inc., La Jolla, CA).

### 2.6. Real-Time PCR

Total RNA was isolated using TRIzol reagent (Sigma-Aldrich) from fresh tissue and RNAprep pure FFPE kit (TIANGEN, DP439) from formalin-fixed tissue, and RNA quality was evaluated with a NanoDrop1000 spectrophotometer (Thermo Fisher Scientific). The first-strand cDNA was synthesized with a PrimeScript RT Master Kit (RR036A; Takara Bio, Shiga, Japan) according to the manufacturer's instructions. Quantitative PCR was performed using a LightCycler 480 SYBR Green I Master (4887352001-1; Roche, Basel, Switzerland) on a LightCycler 480II system (Roche). Reactions were performed in triplicate, and Ct values were calculated using the 2^-ΔΔCt^ method. The expression levels of target genes were normalized to that of GAPDH. Primer sequences are listed in Table 1.

## 3. Results

### 3.1. Dynamic Isl1 Expression in the Human Fetal Retina and hiPSC-Derived Retinal Organoid

**Figure 1:**
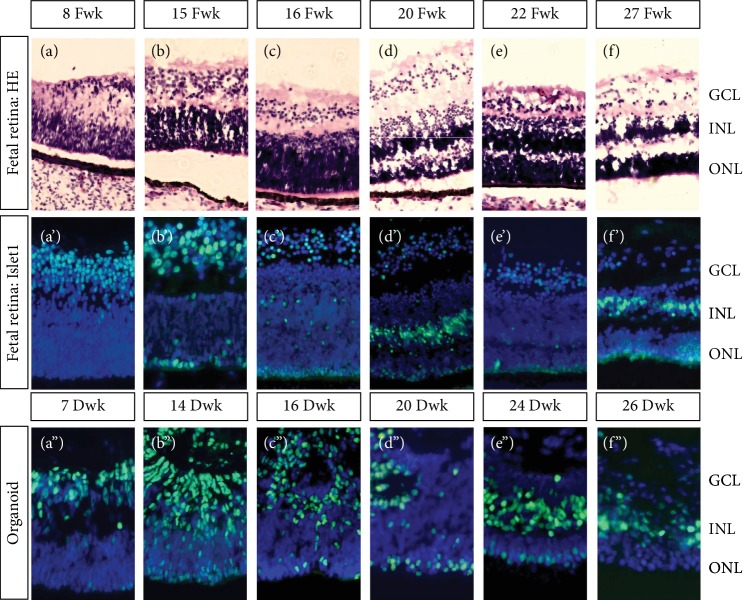
Lamination of human fetal retina and Islet1 dynamic expression in human fetal retina and hiPSC-derived retinal organoid. (a–f) Ganglion cell layer (GCL) first developed in the human fetal retina. Next, the neuroblast layer gradually thickened and divided into an inner nucleus layer (INL) and outer nucleus layer (ONL). (a′–f′) Islet1 was expressed in the GCL in the early stage and then appeared as a monolayer nucleus on the most outer side. Later, sporadic positive nuclei were observed in the thickening neuroblast layer and finally collected on the outer side of the INL. (a^″^–f^″^) Consistent with the human fetal retina, Islet1 was expressed in the GCL first in the retinal organoid. A monolayer of Islet1-positive cells was located on the apical side. In the midperiod, rosettes were detected in the organoid, disturbing lamination. Until late stages, retinal organoid was laminated and Islet1-positive cells were collected in the INL.

Retinogenesis is a dynamic process of cell specification and neuron translocation. As shown in Figures 1(a)–1(f), hematoxylin and eosin staining revealed the lamination and organization of the human fetal retina. Retinal development progressed from the basal side to the apical side. Therefore, retinal ganglion cells (RGCs) were the first cells specified. In the 8^th^ fetal week (Fwk 8), the retinal ganglion cell layer (GCL) became organized, leading to a gap between the GCL and the neuroblast layer. Up to fetal week (Fwk) 16, the neuroblast layer became thicker and a thin gap began to form inside of this layer (Figure 1(c′)). At approximately Fwk 20, the inner nucleus layer (INL) and outer nucleus layer (ONL) were recognized (Figures 1(d) and 1(d′)). Until Fwk 27, the outer plexiform layer (OPL) formed. Dynamic expression of Islet1 protein in the human fetal retina is shown in Figures 1(a′)–1(f′). In Fwk 8, Islet1 was intensively expressed in the GCL. During development, Islet1 expression slowly decreased in the GCL and translocated to the outer layer. Initially, Islet1 was on the most apical side of the retina, followed by the thickening neuroblast layer. Finally, Islet1 was mainly expressed on the apical side of INL. The Islet1 expression pattern in the hiPSC-derived retinal organoid was consistent with that in the fetal retina (Figures 1(a^″^)–1(f^″^)). However, at approximately differentiation week (Dwk) 16, lamination of the retinal organoid was not sufficient, compared to the contemporary fetal retina (Figure 1(c^″^)). In Dwk 24, the Islet1-positive GCL disappeared (Figure 1(e^″^)). Whether this was because the RGCs had matured or disappeared was evaluated by double-staining with RGC markers.

### 3.2. RGC Specification and Islet1 Expression in Human Fetal Retina and Retinal Organoid

**Figure 2:**
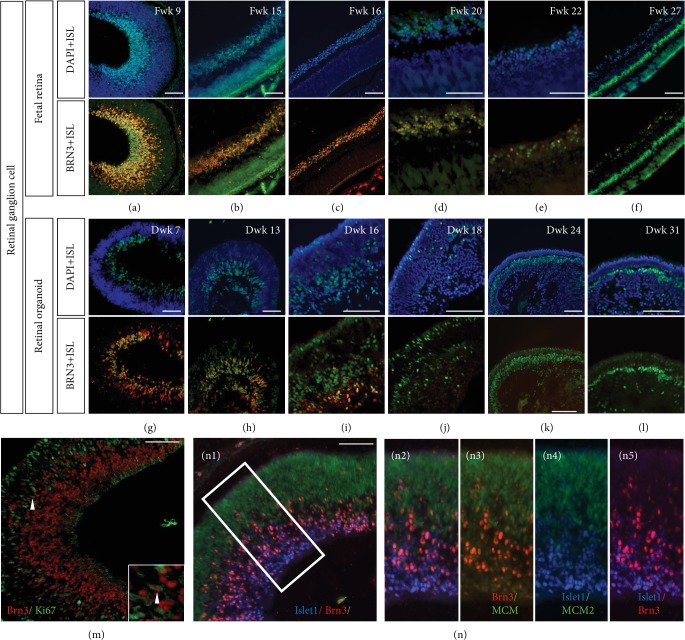
Islet1 coexpressed with Brn3 in retinal ganglion cells. (a–f) Islet1 and Brn3 colabeled retinal ganglion cells in the human fetal retina. From Fwk 20, Brn3 expression gradually decreased and was negative in Fwk 27. (g–i) Islet1-/Brn3-positive retinal ganglion cells were located on the basal side of the retinal organoid but were not laminated well. (j–l) Only sporadic Islet1-positive cells were on the basal side and were Brn3 negative. (m) Most Brn3-positive retinal ganglion cells exited the cell cycle in Fwk 9 retina. The high magnification showed one cell with Brn3 expression but still in the cell cycle. (n1) Triple staining with Islet1, Brn3, and progenitor marker MCM2 in Fwk 9 retina. (n2–n5) Showing high magnification of the white square in (n1) (scale bar = 100 *μ*m).

To determine whether Islet1 colocalizes with early markers of RGC specification, coimmunolabeling with anti-Islet/-Brn3 and anti-Islet/-HuD was performed. In the early weeks, Islet1 was mainly expressed in Brn3-positive retinal ganglion cells. However, at the most basal side, Islet1 expression was higher, while on the outer side, clusters of Brn3-positive but Islet1-negative cells were detected (Figure 2(a)). In the same period of the retinal organoid, Islet1 was colocalized with Brn3 (Figure 2(g)). Consistent with the fetal eye, the innermost cells expressed Islet1 more intensively. During development, the RGC layer was double-stained with Brn3 and Islet1 in the fetal retina (Figures 2(b)–2(e)). However, in the retinal organoid, the Islet1-/Brn3-positive cells did not organize well (Figure 2(i)) and gradually disappeared (Figures 2(j)–2(l)). Unexpectedly, in Fwk 27, the GCL layer Islet-positive cell was not stained by Brn3 antibody (Figure 2(f)). Higher magnification revealed that several Brn3-positive ganglion cells remained in the cell cycle on Fwk 9, expressing Ki67 (Figure 2(m)), while triple staining with MCM2, Brn3, and Islet1 showed that Islet1 levels were higher on the basal side and the progenitor marker MCM2 was on the apical side (Figures 2(n1) and 2(n2)–2(n5)). According to the developmental axis oriented vitreosclerally, the earliest specified ganglion cells were intensively Islet1 positive, while the migrating and specifying RGC were Brn3 positive.

**Figure 3:**
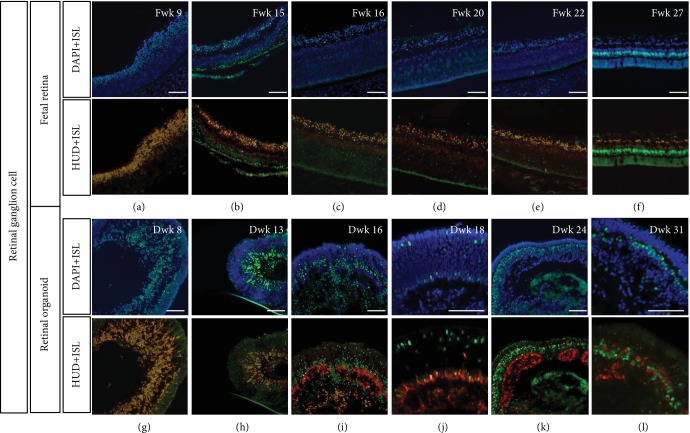
Double immunolabeling of Islet1 and HuD in human fetal retinal and retinal organoid. (a–f) HuD coexpressed with Islet1 in GCL during the early stage of fetal retina development. From Fwk 16, a subpopulation of HuD-positive and Islet1-negative amacrine cells appeared in the INL. (g–l) HuD showed a similar expression pattern in retinal organoids. From Dwk 24, double-positive cells disappeared, and only HuD-single-positive amacrine cells organized as rosettes in the retinal organoid (scale bar = 100 *μ*m).

**Figure 4:**
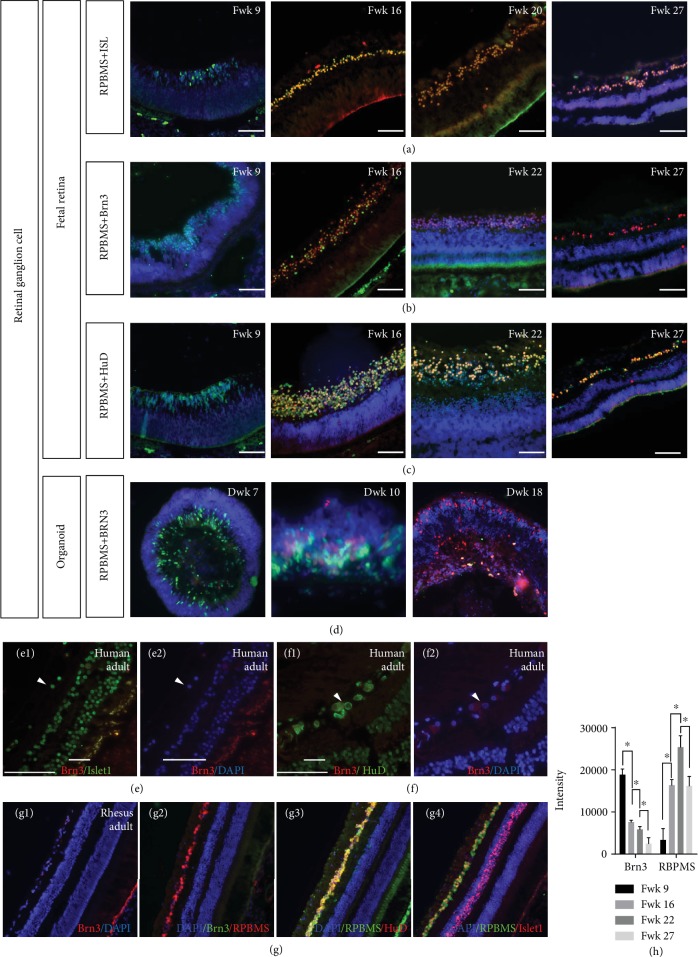
Colabeling RGC with RBPMS, ISL, and Brn3 in human fetal retina, retinal organoid, and rhesus retina. (a) Colabeling RGC with RBPMS and Islet1 in fetal retina. (b) Costaining fetal retina with RBPMS and Brn3. (c) Double staining of HuD and RBPMS in fetal retina. (d) RPBMS expression shared the same pattern in retinal organoids. (e1, e2) In the human adult retina, Islet1 was expressed in retinal ganglion cells (white triangle) but these cells were Brn3 negative. (f1, f2) Human adult ganglion cells were HuD positive (white triangle) but Brn3 negative. (g1) No Brn3-positive cells were detected in rhesus retina. (g2) Retinal ganglion cells were RPBMS positive but could not been stained by Brn3 antibody. (g3) HuD antibody stained retinal ganglion cells along with RBPMS. (g4) Islet1 was positive in both GCL and INL, but RPBMS only labeled retinal ganglion cells. (h) Brn3 immunofluorescence intensity on human fetal retina, along with RBPMS. Scale bar = 100 *μ*m.

Double staining with HuD, which is also an RGC marker as demonstrated by Romero-Aleman and colleagues [16], showed that Islet1 was also coexpressed in the early weeks (Figures 3(a)–3(e)). In Fwk 27, GCL was labeled with Islet1 and HuD, indicating that during this period, the ganglion cell existed but was no longer stained by Brn3 antibody (Figure 3(f)). HuD was positive in the human adult retina (Figure 4(f)). In contrast, the inner side of retinal organoid contained no Islet1-/HuD-positive cells, indicating the disappearance of retinal ganglion cells during late-stage culture (Figures 3(k) and 3(l)).

Costaining with another common RGC marker, RPBMS, it was shown that Islet1, HuD, and Brn3 could be coexpressed in RGC during Fwk 16 to Fwk 22 (Figures 4(a)–4(c)), as well as in retinal organoid (Figure 4(d)). But in the earlier stage (Fwk 8), those Brn3 (+)/HuD (+)/Islet1 (+) RGCs were RPBMS negative, and to Fwk 27, RGCs could only be stained with RPBMS, Islet1, and HuD antibody. To investigate whether the later stage of ganglion cells is still Brn3 positive, we stained the human adult retina with Brn3. As shown in Figures 4(e1), 4(e2), 4(f1), and 4(f2), Islet1-/HuD-positive GCL was Brn3 negative. In rhesus retina, which is one of the most widely used primate models, RGC was also stained by RBPMS, HuD, and Islet1, but not Brn3 (Figures 4(g1) and 4(g2)–4(g4)). Immunofluorescence intensities of Brn3 and RBPMS were evaluated for Fwk 9 to Fwk 27 fetal retina. To Fwk 27, the intensity of Brn3 was extremely low, while that of RBPMS was obviously high (Figure 4(h)).

**Figure 5:**
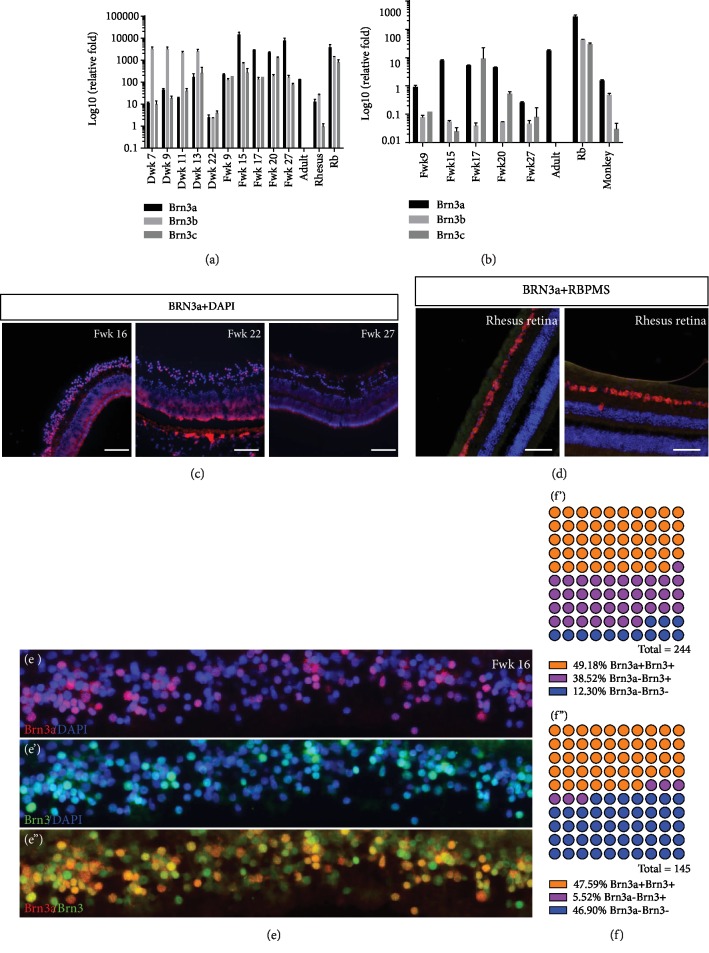
Brn3a expression in human fetal retina, retinal organoid, and rhesus retina. (a) Real-time PCR demonstrated the various expression of Brn3a, b, and c among retinal organoid, fetal retina, adult retina, rhesus retina, and Rb tissue. (b) Considering the rapidly decrease of RGC population in later developmental stage, Brn3a, b, and c expression levels were normalized with RBPMS expression. (c) Brn3a staining in human fetal retina. (d) Costaining of Brn3a and RBPMS in adult rhesus retina. (e–e^″^) Immunostaining of Brn3a and Brn3 in Fwk 16 fetal retina. (f and f′) Cell counting of central (f) and peripheral (f′) Fwk 16 fetal retina.

On the mRNA level, Brn3a expression was significantly low in adult human and rhesus retina as referred to hiPSC. Brn3b and c were not detectable in qPCR in adult human retina. Interestingly, during retinal organoid development, Brn3b expression was the main expression subtype among Brn3a, b, and c, while in human fetal retina, Brn3a was the major (Figure 5(a)). Considering that the retinoblastoma (Rb) cells were regarded as the dedifferentiated cells [17], we included Rb cells as a positive control. In Rb cells, the Brn3a, b, and c mRNA expressions were significantly higher than those in mature retinal cells. During the fetal retina development, RGC population would decrease rapidly in the later stage. Therefore, the expression level of Brn3a, b, and c was then normalized with the expression level of pan-RGC marker, RBPMS. As Figure 5(b) showed, Brn3a expression in both human and rhesus retina was relatively higher after normalization. Further immunostaining of Brn3a was performed on human fetal retina and adult rhesus retina.

In Fwk 16, Brn3a was stained in GCL (Figure 5(c)), and to Fwk 22, the signal obviously decreased. To Fwk 27, there was no Brn3a detected in the retina. Meanwhile, in rhesus retina, the ganglion cells were also RBPMS positive and Brn3a negative (Figure 5(d)). Brn3 and Brn3a costaining was performed on Fwk 16 retina (Figures 5(e)–5(e^″^)). All of the Brn3a-postive cells could be stained with Brn3 antibody, which indicated that the Brn3 antibody did cover those population of Brn3a-positive RGCs. Counting positive cells with ImageJ, the Brn3a-positive cells were the majority of Brn3-positive cells, which was consistent with the results of qPCR (Figures 5(f) and 5(f′)).

### 3.3. Amacrine and Horizontal Cell Development and Islet1 Expression in Human Fetal Retina and Retinal Organoid

Interestingly, in the early stage, HuD protein was expressed mainly in the cytoplasm (Figures 3(g) and 3(h)). From Fwk 15, a layer of HuD-positive/Islet1-negative cells was observed (Figure 3(b)) and HuD was in the nucleus in these cells. These cells were more obvious in the Dwk 16 retinal organoid (Figure 3(i)) and settled between the retinal ganglion cells and outer Islet1-single-positive cells. Based on the cells on the inner side of INL and previous studies showing that HuD protein was also expressed in amacrine cells [16], this cluster of cells may be a subtype of amacrine cells. Until Fwk 27, HuD-positive cells lined up on the inner side of the INL, apart from the Islet1-positive cells (Figure 3(f)). However, in the retinal organoids, these cells remained mixed with Islet1-positive cells in the INL (Figure 3(l)).

**Figure 6:**
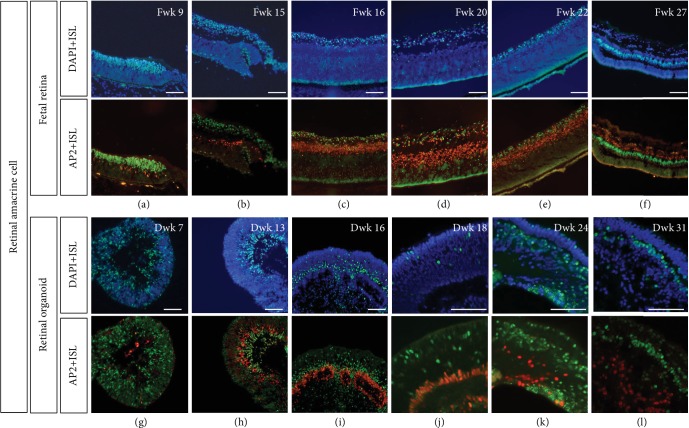
Islet1 expression and postmitotic amacrine marker, AP2*α*. (a–f) Amacrine cell developed as early as Fwk 9 and then organized in the inner side of the INL, without coexpression of Islet1. (g–l) In the retinal organoid, AP2*α*-positive amacrine cell was observed at Dwk 13, later than in the fetal retina. Until Dwk 16, the number of amacrine cells obviously increased but was organized as rosettes. Until Dwk 31, the AP2*α*-positive cells showed a disorderly arrangement (scale bar = 100 *μ*m).

AP2*α*-positive cells were also located in the INL. According to a study by Bassett et al., AP2*α* is exclusively expressed in postmitotic amacrine cells during retinal development and in mature amacrine cells in the adult retina [18]. From Fwk 9, several postmitotic amacrine cells were observed in the fetal retina (Figure 6(a)), but not observed in the retinal organoid until Dwk 13 (Figure 6(h)). These subpopulations of amacrine cells shared similar location of HuD-positive/Islet1-negative cells (Figures 6(i)–6(k)).

Prox1, a developing horizontal cell marker, colocalized with Islet1 (Supplementary [Supplementary-material supplementary-material-1]). Further, the absence of colocalization of Islet1 with Prox1 at Fwk 22 in the human fetal retina confirmed that Islet1 is not expressed in mature but rather in developing horizontal cells (Supplementary [Supplementary-material supplementary-material-1]). Prox1 highlights horizontal, bipolar, and amacrine cells in the adult retina. Its expression showed a high degree of colocalization with Islet1 signal along the outermost border of the INL where Prox1 identifies bipolar cells.

### 3.4. Bipolar Cell Development Was Delayed in Retinal Organoid

**Figure 7:**
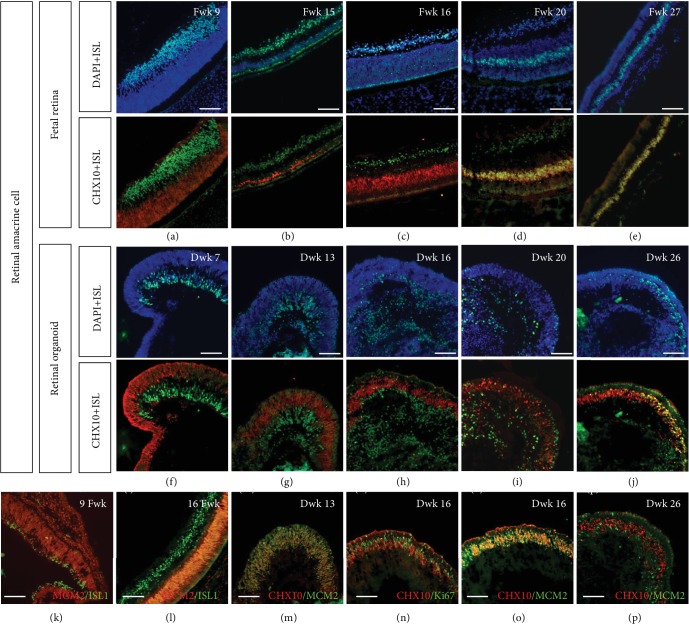
Coimmunolabeling of Islet1 and CHX10 in human fetal retinal and retinal organoid. (a–c) CHX10 did not colocalize with Islet1 in the early stage, indicating CHX10 is a retinal progenitor marker during this period. (d, e) Layer of double-positive bipolar cells settled in the INL in the fetal retina. (f–i) CHX10-labeled retinal progenitors in the neuroblast layer in retinal organoids. (j) Some CHX10-positive cells colabeled with Islet1. (j, k) Another neural progenitor marker, MCM2, revealed undifferentiated cells in the fetal retina. (m–o) MCM2 and cell cycle marker, Ki67, confirmed that CHX10 was expressed in progenitors in this period rather than in bipolar cells. (p) To Dwk 26, CHX10-positive bipolar cells were MCM2 negative (scale bar = 100 *μ*m).

In early development, CHX10 (also known as VSX2) is expressed in retinal progenitor cells. As shown in Figures 7(a)–7(c) and 7(f)–7(h), in the early stage, CHX10-positive progenitor cells were in the neuroblast layer and were Islet1 negative. From Fwk 20, a cluster of cells coexpressing CHX10 and Islet1 was located in the inner nucleus layer (Figures 7(d)–7(e)). According to previous studies, in later developmental stages, as progenitor cells differentiate and exit the cell cycle, CHX10 is expressed only in bipolar cells and is regarded as a pan-bipolar marker [7, 19, 20]. Interestingly, in retinal progenitor cells, the CHX10-positive nucleus was oblong (Figures 7(f)–7(i)), while in bipolar cells, it was oval (Figure 7(j)). However, in the retinal organoid, the colabeled cells did not develop until Dwk 26, which is delayed compared to in the fetal retina (Figure 7(j)). To confirm whether CHX10-positive cells were progenitor or bipolar, we double stained the cells with CHX10 and MCM2, another progenitor marker [21]. At Dwk 13 and 16, nearly all CHX10-positive cells were found to be progenitors (Figures 7(m)–7(o)), but the inner side population of cells had already exited the cell cycle. At Dwk 26, CHX10-/Islet1-positive bipolar cells were not stained with MCM2 (Figure 7(p)). PKC*α* identified rod bipolar cells [22]. In the retinal organoid, the PKC*α*-/Islet1-positive bipolar cells were in the INL (Supplementary [Supplementary-material supplementary-material-1]).

### 3.5. Islet1 Expression in the Photoreceptor Cell Layer

**Figure 8:**
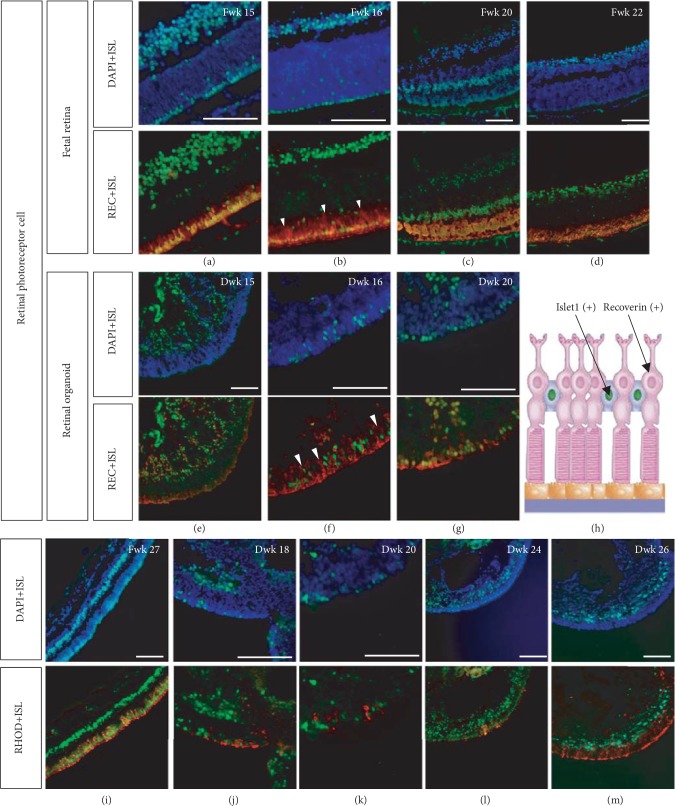
Isl1 expression in developing photoreceptor cells. (a–d) A monolayer of Islet1 immunoreactive nuclei was observed from Fwk 15; the cells were Recoverin positive. However, as indicated by the white triangle, some Recoverin-positive cells were Islet1 negative, and their nuclei were located on the inner side to Islet1-positive cells. (e) In Dwk 15, Recoverin-positive developing photoreceptors were migrating from the basal side to the apical side. (f) Similar to in the fetal retina, some developing photoreceptors with inner arranged nuclei were Recoverin positive but Islet1 negative. (g–h) Developing photoreceptors in the retinal organoid. (i) In Fwk 27, cells with inner arranged nuclei were Rhodopsin-positive rods, and the Islet1-nuclei were between the soma and inner segment of rods as the schematic diagram (h). (j–m) Rod differentiation in retinal organoids (scale bar = 100 *μ*m).

At Fwk 15, a mononucleus layer of Islet1-positive cells was observed. When double labeled with Recoverin, some cells were colocalized (Figures 8(a)–8(d)). Additionally, a population of Recoverin-positive cells that was Islet1 negative was detected, with the nucleus lined up on the inner side of the double-positive photoreceptors (Figure 8(b), white triangle). A similar pattern was observed in the retinal organoid; however, in Dwk 15, Recoverin-positive developing photoreceptors were still migrating from the basal side to the apical side, indicating a slight delay in retinal organoid development compared to in the fetal retina.

Double staining with Rhodopsin and Islet1 revealed that the Islet1-negative photoreceptors were rods (Figures 8(i)–8(m)). The Islet1-positive nuclei were between the soma and the inner segment of the rods (Figure 8(h)). Double staining of Islet1 and cone markers (S-opsin and L/M-opsin) in retinal organoid demonstrated that cone cells were Islet1-positive (Supplementary [Supplementary-material supplementary-material-1]). However, when labeling the fetal retina and rhesus retina, no obvious colocalization was seen (Supplementary [Supplementary-material supplementary-material-1]). The peripheral retina was Islet1 positive in the most outer layer (Supplementary [Supplementary-material supplementary-material-1]), but in the central retina, when cone markers expressed, Islet1 expression decreases (Supplementary [Supplementary-material supplementary-material-1]). Developing photoreceptors are well-organized by Fwk 15, but the inner segments did not develop until Fwk 16. Until Fwk 20, the inner segment could easily be detected. However, in the retinal organoid, when the inner segment appeared in Dwk 16, a cluster of immature photoreceptors migrating from the basal side remained.

## 4. Discussion

We evaluated the dynamic expression of Islet1 in the human fetal retina and compared the hiPSC-derived retinal organoid with human fetal retina development. The results revealed that Islet1 temporal and spatial expression was generally similar to that in various vertebral animal models. However, we also found the Islet1 was specifically expressed in cone precursors of the human fetal retina. Further, the retinal organoid showed the same patterns of Islet1 expression as the fetal retina, indicating its potential as a development model and drug-screening model. However, in later stages of development, the retinal organoid showed a developmental delay compared to the human fetal retina in the specification of photoreceptors and retinal lamination.

A recent study by Bejarano-Escobar et al. [6] confirmed the presence of the LIM-domain transcription factor Isl1 in differentiating and mature ganglion, amacrine, bipolar, and horizontal cells in the retina of mammals, birds, reptiles, fish, and *Xenopus laevis*. In the present study, we examined the retina of human embryos. The Islet1 spatiotemporal expression in human fetal showed a very similar pattern to in mammals, from ganglion to amacrine and bipolar cells. Further, the “retina-in-dish” showed a similar pattern of expression.

Consistent with a study by Prasov and Glaser [23], human fetal retinal ganglion cells coexpressed Brn3 and Islet1 during retinal development. Interestingly, in the fetal retina, the signal intensity between Brn3 and Islet1 varied from the basal side to the apical side. In the most inner side, cells showed stronger expression of Islet1 than of Brn3. This layer of cells was postmigrated RGCs, which were the first specified retinal neurons. In the outer direction from this layer, cluster cells expressed the two proteins in the same quantities. Further towards the outer side of the neuroblast layer, migrating RGCs showed higher expression of Brn3. A similar pattern was reported mouse models [24]. Meanwhile, Li and colleagues' study also shown that the Islet1 and Brn3 (including Brn3a, b, and c) collaborated nonsynergistically in regulating RGC differentiation [25]. In the retinal organoid, this phenomenon was also observed. However, the postmigrated RGC with higher Islet1 levels did not line up as clearly as in the fetal eye. Thus, Islet1 indicated more mature RGCs, while Brn3 indicated relatively immature RGCs.

We found that Brn3 expression gradually decreased during fetal development. The RGC in adult human retina could not be stained by Brn3 antibody, as in the adult primate retina. Then, we investigated the mRNA level of Brn3a, b, and c during the development of fetal retina and adult retina. In adult retina, Brn3b or c was not detectable, while Brn3a was 135-fold higher, consistent with Whitmore's study by RNA-seq that POU4F1 (Brn3a) was enriched in the macular part of retina [26]. After normalized with another pan-RGC marker, RBPMS, the Brn3a level was even higher in adult human retina. Costaining Brn3 and Brn3a in fetal retina indicates that the Brn3 antibody we used could cover those population of Brn3a-positive RGCs. Therefore, although the Brn3a transcript factor was high in adult retina, there is a possibility that only a few of them translated into protein, leading to the negative result in immunofluorescence. Another research performing RNA-seq with human retina showed that the Brn3b (POU4F2) was detectable [27], although the expression level was relatively much lower. Considering the RNA degradation in PFA-fixed sample, this could be the reason that Brn3b was not detected in the present study. In fetal retinas, Brn3a expression was gradually decreasing along development. Those periods with a high level of Brn3a expression coincidently matched with the period of RGC redistribution in retinal development [28–30]. As a control, the dedifferentiated retinal cells, retinoblastoma cells expressed a high level of Brn3a, b, and c, indicating the relationship between low level Brn3 and retinal neuron maturation. Contrarily, the Brn3a level kept low in retinal organoid development and they mainly expressed a high level of Brn3b. Consistent with Langer et al.'s findings, hiPSC-derived RGC population had subtype profile varied from human RGCs [31]. This result indicated that although the stem cell-derived RGC was highly similar to human RGCs in morphology, even in electrophysiological characteristics, they were still in high heterogeneity. Considering the relation between Brn3a and the bcl-2-related apoptosis pathway [32, 33], the varied transcript profile of Brn3a, b, and c in stem-cell RGC may also be one of the factor that related to the gradually RGC apoptosis in long-term culture of retinal organoid. Because most studies of RGC development [34–38] and RGC damage in glaucoma [39, 40] used Brn3 as a marker in rodent models, caution should be used when extrapolating the results to primates and stem cell-derived retinal neurons.

Moreover, HuD protein (also known as ELAVL4) is an RGC marker [15]. In contrast to Brn3, HuD is expressed in the GCL in the mature retina. Additionally, in the present study, after Fwk 15 and Dwk 16, HuD was expressed on the inner side of the GCL, which was found to contain amacrine cells. This agrees with the results of Ekstrom and Johansson [41].

Correspondingly, double labeling of Islet1 and amacrine- and bipolar-specific markers in the human fetal retina and retinal organoid showed the same pattern as in the mouse retina, chicken retina, etc. [7, 42–44]. Meanwhile, Islet1 also expressed in amacrine cells and bipolar cells in pig, and they appeared around midgestation [45], which was consistent with fetal retina. Although AP2*α*-positive amacrine cells in retinal organoids gained their fate during a similar period as in the human fetal retina, they failed to migrate and organize in later development. In contrast, the development of bipolar cell delayed, but the cells organized into a layer in INL.

Few studies have focused on Islet1 and photoreceptor specification. According to Wang et al., Islet1-/Recoverin-positive cells were cone precursors [46], which were labeled by L/M-opsin in later development. Moreover, a review by Bejarano-Escobar et al. described that most studies showed that Islet1 failed to colocalize with typical markers of rods and cones [6]. In the present study, we found that in the ONL of the human fetal retina, Islet1 labeled Islet1-/Recoverin-positive cone precursors. Moreover, in later development stages, when we stained cone cells with S-opsin and L/M-opsin antibody, no colabeling was seen with Islet1 in human fetal retina and rhesus retina. But in long-term cultured retinal organoid, S-opsin or L/M-opsin was colabeled with Islet1 in cone cells. Meanwhile, we also observed that from peripheral to central fetal retina, Islet1 downregulated in the most outer layer coincided with cone-opsin upregulation. Fischer et al.'s study demonstrated that the upregulation of cone-opsin coincided with the downregulation of Islet2 in photoreceptors in the far peripheral regions of retina [9]. This may be one of the reasons why no coexpression of cone-opsin and Islet1 was observed in fetal retina and rhesus retina.

In conclusion, this is the first study to describe the Islet1 and Brn3 expression pattern throughout development in the human fetal retina. The expression of Islet1 in subsets of retinal neurons was mainly consistent across species from fish to human, supporting that Islet1 plays a critical role in human retinal cell specification, differentiation, and maintenance. Additionally, we determined the full picture of development and cell specification in hiPSC-derived retinal organoid comparing to in the fetal retina.

There were some limitations to this study. First, although Brn3 and Brn3a did not stain RGCs in the adult primate retina, whether Brn3b and c proteins were all covered by present antibody was not clear. Due to the shortage of samples, quantification of protein was not able to be performed in the present study. Thus, it was unclear how many Brn3 transcript factors would translate into protein. Therefore, the role of Brn3 and Islet1 in RGC differentiation in primate requires further analysis. Second, a previous study demonstrated that the Islet coexpressed with cone markers was Islet2, rather than Islet1 [9]. Thus, although the Islet1 primary antibody used was monoclonal, further confirmation should be performed by RNA *in situ* hybridization. Finally, compared to the fetal retina, the hiPSC-derived retinal organoid has three main limitations: RGC was not preserved in later development, RGC transcript profile differed from those in fetal retina, and the lamination and organization were not distinct. Therefore, the differentiating protocol must be optimized, possibly by using additional transcription factors.

## Figures and Tables

**Table 1:** Primer used in real-time PCR.

Gene	Forward	Reverse
GAPDH	TGCACCACCAACTGCTTAGC	GGCATGGACTGTGGTCATGAG
Brn3a	ACCACCATTATTACCACCTCCC	CTCGCTCGTTTGGTTTTCGTT
Brn3a (rhesus)	ACCACCATTATTACCACCTCCC	CCGGCTCATTTGGTTTTCGTT
Brn3b (rhesus)	CAAGCAGCGACGCATCAAG	GGGTTTGAGCGCGATCATGTT
Brn3b	CCATGAACCCCATGCACCA	CATGCAGCCCATGTGCGA
Brn3c	CATGGGCATGAGTCACCCG	GTCTGACTCCACGTCGCTGA
RBPMS	CGAGAAGGAGAACACCCCGA	AGGCCACTGACAAATAGGGTC
RBPMS (rhesus)	CGAGAAGGAGAACACCCCGA	AGGCCACTAACAAATAGGGTC

## Data Availability

The immunofluorescence data used to support the findings of this study are included within the article and the supplementary information file.

## Supplementary Materials

Supplementary Materials Supplementary Figure 1: Islet1 expression in developing horizontal cells. (A) In Fwk 16, Islet1 and Prox1 were co-immunoreactive in INL. (B–C) In later stages, Prox1-positive cells were in the outer side of the INL, without double-staining with Islet1." "Supplementary Figure 2: Bipolar cells expressing CHX10 and PKC*α* in human fetal retina and retinal organoid. (A) In Dwk 20, PKC*α* and CHX10-positive cells collected on the basal side of the retinal organoid. (B–C) Rod bipolar cells began to line up in the INL. (D) In this period, the cells were relatively well-organized." "Supplementary Figure 3: Co-immunostaining with Islet1 and cone markers. (A) Islet1 and L/M-opsin co-expressed in Dwk 31 retinal organoid. (B) In Dwk 31 retinal organoid, Islet1 and Sopsin did not co-label in cone cells. (C) In Dwk 36, cone cell was double-stained with S-opsin and Islet1. (D-F) Islet1 mainly expressed in INL of Fwk 27 fetal retina and adult rhesus retina, and the cone cells was Islet1-negative. (G) In this period, the monolayer of Islet1-positive cells still existed in the peripheral retina. (H-I) However, Islet1 expression gradually decreased from the peripheral to central areas, while S-opsin and L/M-opsin increased. In the central retina, the outer layer cells were Islet1-negative and rich in cone markers.
