# Supplementary material for: Islet1 and Brn3 Expression Pattern Study in Human Retina and hiPSC-Derived Retinal Organoid
Source: Stem Cells Int. 2019 Dec 10;2019:8786396. doi: 10.1155/2019/8786396 (PMC6925930; doi:10.1155/2019/8786396)
Supplement: Supplementary Materials — Supplementary Figure 1: Islet1 expression in developing horizontal cells. (A) In Fwk 16, Islet1 and Prox1 were co-immunoreactive in INL. (B–C) In later stages, Prox1-positive cells were in the outer side of the INL, without double-staining with Islet1." "Supplementary Figure 2: Bipolar cells expressing CHX10 and PKCα in human fetal retina and retinal organoid. (A) In Dwk 20, PKCα and CHX10-positive cells collected on the basal side of the retinal organoid. (B–C) Rod bipolar cells began to line up in the INL. (D) In this period, the cells were relatively well-organized." "Supplementary Figure 3: Co-immunostaining with Islet1 and cone markers. (A) Islet1 and L/M-opsin co-expressed in Dwk 31 retinal organoid. (B) In Dwk 31 retinal organoid, Islet1 and Sopsin did not co-label in cone cells. (C) In Dwk 36, cone cell was double-stained with S-opsin and Islet1. (D-F) Islet1 mainly expressed in INL of Fwk 27 fetal retina and adult rhesus retina, and the cone cells was Islet1-negative. (G) In this period, the monolayer of Islet1-positive cells still existed in the peripheral retina. (H-I) However, Islet1 expression gradually decreased from the peripheral to central areas, while S-opsin and L/M-opsin increased. In the central retina, the outer layer cells were Islet1-negative and rich in cone markers. [file 8786396.f1.pdf]

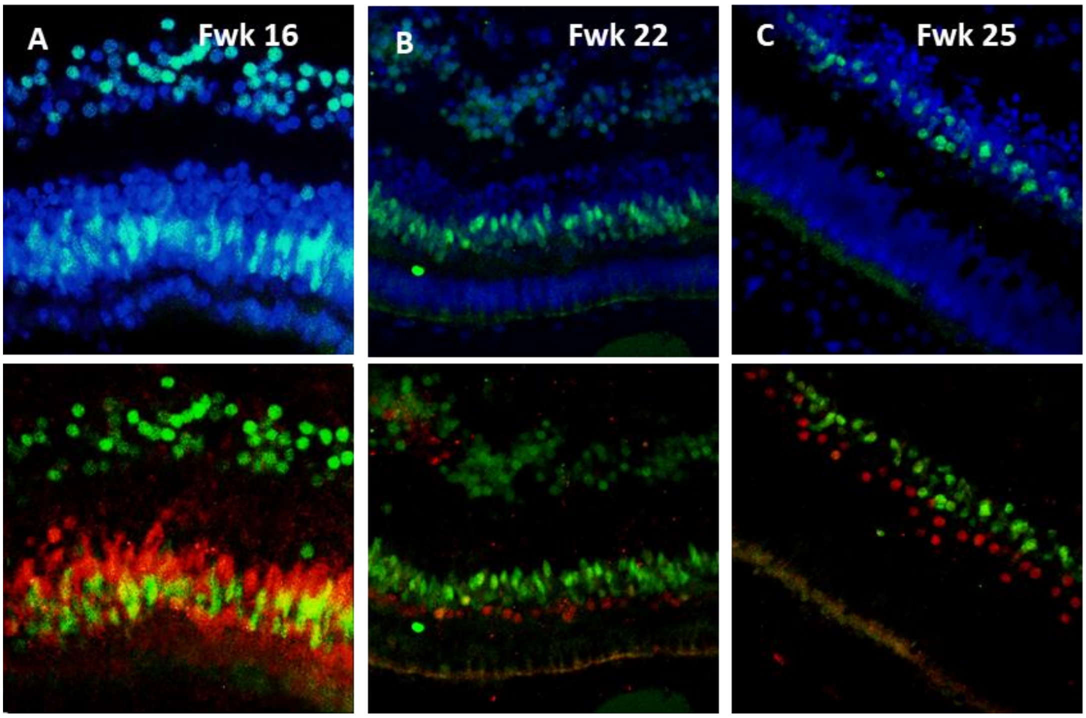

Supplementary Figure 1. Islet1 expression in developing horizontal cells. (A) In Fwk 16, Islet1 and Prox1 were co-immunoreactive in INL. (B–C) In later stages, Prox1-positive cells were in the outer side of the INL, without double-staining with Islet1.

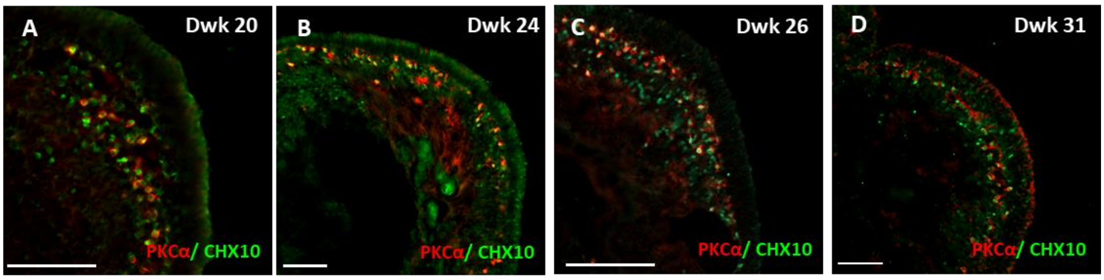

Supplementary Figure 2. Bipolar cells expressing CHX10 and PKCα in human fetal retina and retinal organoid. (A) In Dwk 20, PKCα and CHX10-positive cells collected on the basal side of the retinal organoid. (B–C) Rod bipolar cells began to line up in the INL. (D) In this period, the cells were relatively well-organized.

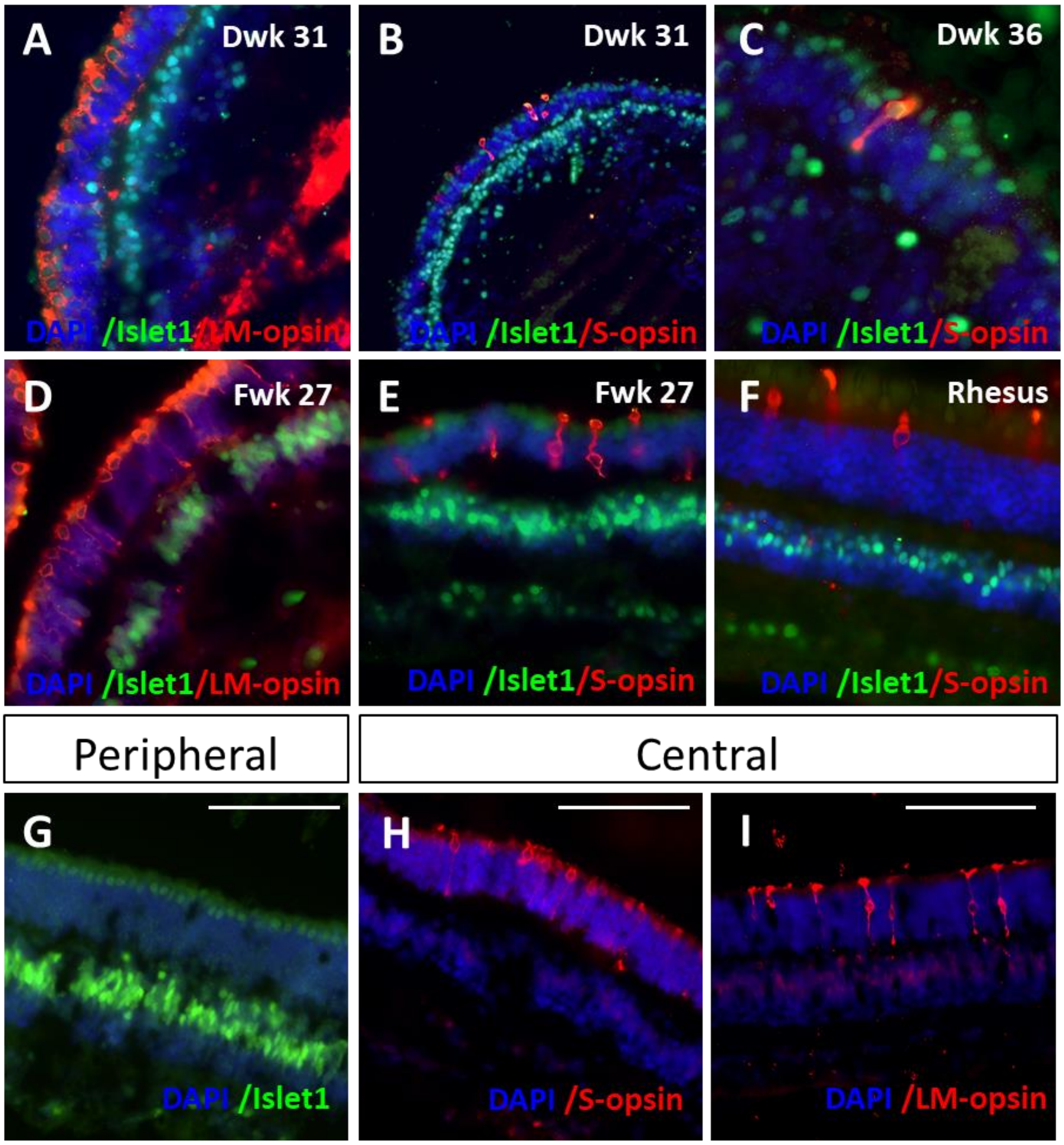

Supplementary Figure 3. Co-immunostaining with Islet1 and cone markers. (A) Islet1 and L/M-opsin co-expressed in Dwk 31 retinal organoid. (B) In Dwk 31 retinal organoid, Islet1 and S-opsin did not co-label in cone cells. (C) In Dwk 36, cone cell was double-stained with S-opsin and Islet1. (D-F) Islet1 mainly expressed in INL of Fwk 27 fetal retina and adult rhesus retina, and the cone cells was Islet1-negative. (G) In this period, the monolayer of Islet1-positive cells still existed in the peripheral retina. (H-I) However, Islet1 expression gradually decreased from the peripheral to central areas, while S-opsin and L/M-opsin increased. In the central retina, the outer layer cells were Islet1-negative and rich in cone markers.
